# The Prairie State Veterinary Colleges

**Published:** 1898-06

**Authors:** 


### THE PRAIRIE STATE VETERINARY COLLEGES.

The Chicago Veterinary College was established in 1883. It is chartered by the State. Dr. R. J. Withers still holds the position of President of the Board of Trustees.

Dr. A. H. Baker fills the important chairs of professor of theory and practice, surgery, obstetrics, and physiology.

Dr. James Henderson, who recently left Chicago for Scotland, filled the chairs of cattle-pathology and meat- and milk-inspection.

The school graduated its 15th class this year. It has now 545 graduates in active practice. Some 35 of her graduates have held or are holding official positions in connection with State colleges, State and National veterinary sanitary and inspection work; 10 of her graduates have died since the school opened.

The school is a private institution, being owned entirely by three members of the faculty. It for many years maintained a course of two years of six months each, but for the past three years the curriculum has been extended to require three years of six months each.

The McKillip Veterinary College was chartered in 1892. This school is also a private institution, maintained and owned by its founder, Dr. M. H. McKillip. Its buildings are splendidly arranged with ample laboratories for teaching, and its facilities for conveying practical knowledge unsurpassed by any of the schools. The clinical facilities afforded are unusually good, and in extent cover one of the largest private practices in this country.

From its inception this school has maintained a three-years' course of six months each. Its first course of instruction was give in the fall of 1894. About fifty students have matriculated at the college since its opening. The first class of graduates, numbering ten, were sent forth in 1897, while the list of graduates for 1898 number nine. The practical staff of instructors of the institution are five in number.

The State University, at Champaign, maintains a veterinary chair of instruction, which is filled by Dr. D. J. McIntosh. The usual short course of veterinary science intended for agricultural students is maintained. During the past year there has been much agitation looking to the establishment of a veterinary department of this university. It has been strongly urged by such influential papers as the *Breeders' Gazette*, and the State Legislature has been urged to make the necessary appropriation. Such an institution would, no doubt, be given greater security so far as perpetuation is concerned, but many Western State universities are so thoroughly under political control and direction that able and efficient corps of instructors are difficult to obtain and hard to retain under such conditions. We should be glad to see one of the schools of Chicago identified with the Chicago or Northwestern University, where their more complete and enlarged maintenance would be doubly assured without wholly depending upon their incomes from students' fees to gauge the course of instruction. Such a department would no doubt in time be largely endowed by some of those who have grown so rich in the various branches of the livestock industry, so largely contributed to by the veterinary profession, and whose successful maintenance and advance grows daily more dependent upon the practitioners of comparative medical science. We trust that the agitation already started will continue until this much-to-be-desired end is assured.

